# Microfluidic Patterning of Metal Structures for Flexible Conductors by In Situ Polymer‐Assisted Electroless Deposition

**DOI:** 10.1002/advs.201600313

**Published:** 2016-11-01

**Authors:** Suqing Liang, Yaoyao Li, Tingjiao Zhou, Jinbin Yang, Xiaohu Zhou, Taipeng Zhu, Junqiao Huang, Julie Zhu, Deyong Zhu, Yizhen Liu, Chuanxin He, Junmin Zhang, Xuechang Zhou

**Affiliations:** ^1^ College of Chemistry and Environmental EngineeringShenzhen UniversityShenzhen518060P. R. China; ^2^ Department of ChemistryThe Chinese University of Hong KongShatinN.T., Hong Kong SARP. R. China

**Keywords:** electroless metal deposition, flexible electronics, microfluidics, polymer brushes, surface patterning

## Abstract

**A low‐cost, solution‐processed, versatile, microfluidic approach** is developed for patterning structures of highly conductive metals (e.g., copper, silver, and nickel) on chemically modified flexible polyethylene terephthalate thin films by in situ polymer‐assisted electroless metal deposition. This method has significantly lowered the consumption of catalyst as well as the metal plating solution.

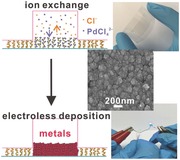

Flexible electronics, electronics that can function under mechanical deformation, is in high demand toward a wide variety of new applications, including wearable electronics, flexible displays, electronic skins, energy storage, medical implants, sensors, and biological actuators.^1^, ^2^, ^3^, ^4^, ^5^, ^6^, ^7^, ^8^, ^9^, ^10^, ^11^, ^12^ To realize the function of these electronic devices, highly conductive electrodes, contacts, and interconnects with large mechanical flexibility (e.g., bending, folding, stretching, compressing, and twisting) are considered as one of the most important components. Remarkable development of the fabrication strategies has been made in the past few years. Among various organic and inorganic conductive materials, metals are recognized as promising materials for the fabrication of flexible conductors, owing to their extreme high conductivity and stability. Hence, the engineering of well‐defined metal structures (or thin films) on flexible substrates has attracted much attention, and numerous advances have been achieved by applying various metallization methods, such as physical vapor deposition,^13^ chemical vapor deposition,^14^ electrochemical plating, and electroless metal deposition (ELD).

Among the above‐mentioned methods, ELD is known as a versatile, site‐selective, solution‐processed, low‐cost, and scalable method, which renders this method particularly suitable for fabricating metal structures on flexible plastic substrates at low temperatures.^15^ More recently, several advances in polymer‐assisted ELD^16^, ^17^, ^18^ have been accomplished for the fabrication of highly conductive metal structures for flexible and stretchable interconnects,^19^, ^20^, ^21^, ^22^ supercapacitors,^23^, ^24^ conductive textiles,^25^, ^26^ and optoelectronic devices.^27^, ^28^ The polymer‐assisted ELD typically involves three major steps: surface modification of functional polymer anchoring layers, loading of catalyst moieties to the polymer anchoring layer by ion exchange, and site‐selective metal electroless deposition. Despite those fruitful advances, ELD processes typically require a bulk of metal plating solution as well as catalyst (e.g., palladium‐based catalyst), which dramatically increases the cost of production. To lower the catalyst consumption, a matrix‐assisted catalytic printing for ELD was developed by the Zheng and co‐workers.^29^ Similarly, Zhang et al. approached the high cost by printing of catalytic inks on papers for ELD.^30^ Alternatively, Garcia et al. developed a nonexpensive catalyst (palladium‐free) strategy for ELD to further lower the cost of fabrication.^31^ However, an ELD method still requires large amounts of metal plating solution. On the other hand, it is known that microchannels have been widely used in surface patterning of various materials, including metals.^32^, ^33^ For example, Goluch et al. reported the fabrication of metal thin film structures on curved surfaces by electroless deposition via a polydimethylsiloxane (PDMS) patch of microchannel.^34^ Taking into account the high quality of metal structures prepared by the polymer‐assisted electroless metal deposition, it is of significance to develop a microfluidic patterning technique to further lower the cost of production without changing the high performance of conductivity and mechanical durability.

In this Communication, we for the first time report a low‐cost, solution‐processed, and microfluidic approach for patterning metal structures on chemically modified polyethylene terephthalate (PET) thin films by in situ polymer‐assisted ELD. In this method, a PDMS stamp consisting of arrays of microchannels was utilized to consequently load the catalyst and the electroless plating solutions to the PET substrate, which was pre‐modified with a thin layer of poly[2‐(methacryloyloxy)ethyl‐trimethylammonium chloride] (PMETAC). Thus, the loading of the catalyst by ion exchange and coating of metals by in situ ELD were performed consequently within the microchannels, leading to the formation of patterned metal structures on PET substrates. To validate this method, we have successfully fabricated patterns of copper, silver, and nickel on PET substrates with extreme high electrical conductivity and excellent mechanical flexibility. Our results show that the conductivity of the as‐made metals is comparable to that of bulky metals, i.e., 3.0 × 10^7^, 5.2 × 10^7^, and 1.0 × 10^7^ S m^−1^, for Cu, Ag, and Ni, respectively, which is among the best for metals prepared by ELD. Remarkably, the as‐made metal‐PET exhibits a stable conductivity even upon a large number of bending cycles (e.g., 5000) at different bending curvatures. We further demonstrate that the as‐made metal‐PET composites are capable for long‐term applications, such as flexible conductors and their flexible LED circuits.

The key novelty of this method is that both the loading of catalyst and ELD process are carried out in situ with the aid of microchannels, which leads to two superior characteristic features. The most obvious feature is the total consumption of the catalyst and metal plating solutions, which significantly decreases expenses. For instance, to guarantee the completion of ion exchange, a large quantity of catalyst solutions is applied in traditional experiments. However, an ultrasmall quantity of catalyst ions is immobilized onto the functional groups of the PMETAC on the patterning area. The present study signifies that excess catalyst solutions can be readily recycled and thus the total consumption of catalyst is dramatically decreased, which is much different from that of our previous studies, which were conducted via directly printing excess amount of catalyst solution onto the substrate. Simultaneously, the total consumption of metal plating solutions also decreases. The less obvious, yet more important characteristic feature is that both the whole ion exchange and the ELD process are scaled down and confined to the microchannels, which significantly promotes the reactions. As a result, a much shorter duration for the ion exchange and ELD process is required as compared to our previous studies, which prepared bulky metal thin films as well as patterned structures by catalytic printing.^29^

The fabrication process of the metal‐patterned PET is schematically illustrated in **Figure** **1**a. The surface modification of PET (50 µm thick) with PMETAC polymers was carried out according to the procedures described in our previous study.^25^, ^35^ In a typical experiment, the PET substrate was first treated with air plasma for 5 min, which was followed by coating a layer of vinyltrimethoxysilane (VTMS) via silanization (Figure S1, Supporting Information). The resulting VTMS‐PET substrate was then treated with a polymerization solution containing an METAC monomer and a potassium persulfate (KPS) initiator for a certain time, leading to the fabrication of PMETAC‐PET substrate. The successful surface modification at the above steps can be verified by consequent measurements of the water contact angle of the PET substrate. Indeed, the contact angles differ for the PET, air plasma treated‐PET, VTMS‐PET, and PMETAC‐PET, and were determined to be 75 ± 10°, 6 ± 5°, 75 ± 5°, and 17 ± 5°, respectively (Figure S2, Supporting Information). To carry out the patterning of metals, a patch of PDMS microchannel was reversibly attached to the as‐made PMETAC‐PET substrate. Then a catalyst solution was infused into the microchannel for 3 min at a flow rate of 0.1mL min^−1^ via a peristaltic pump. During this process, the [PdCl_4_]^2−^ catalytic ions were immobilized by ion exchange onto the quaternary ammonium groups of PMETAC polymer chains. Afterward, deionized (DI) water was infused into the microchannel to rinse the physically adsorbed catalyst. With this method, Cu‐PET was fabricated by applying a typical Cu electroless plating solution in the microchannel for 2 min at the flow rate of 0.1mL min^−1^. Afterward, the channel was rinsed by DI water and finally the microchannel was removed, resulting in a Cu‐patterned PET substrate. Figure 1b–d shows optical micrographs of the PDMS microchannel and the as‐made Cu‐PET substrate. To further evaluate this patterning method, Cu‐PET patterns with width varying from 50 to 250 µm were fabricated (Figure S3, Supporting Information). Indeed, a bendable Cu‐patterned PET substrate was obtained, which was completely reproduced from the corresponding microchannels.

**Figure 1:**
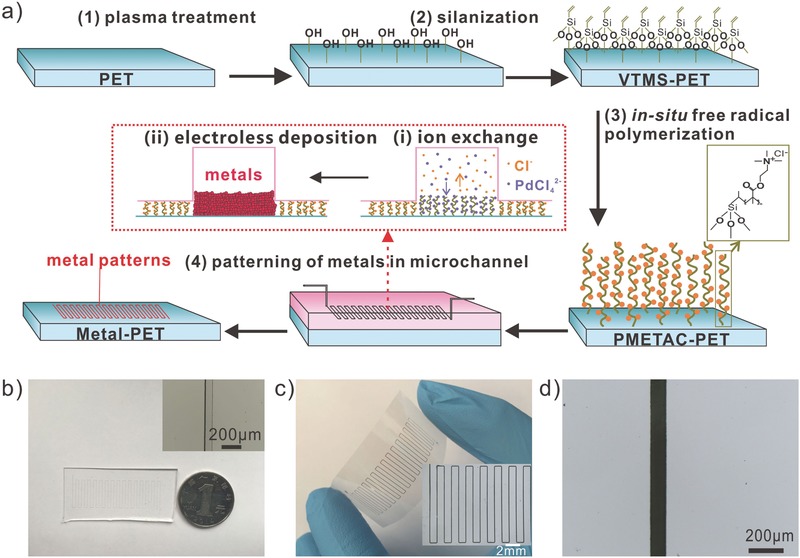
a) Schematic illustration of the fabrication process of the metal‐patterned PET substrate. First, polyelectrolyte (PMETAC) brushes are fabricated by means of in situ free‐radical copolymerization. Subsequently, a PDMS stamp of microchannel is attached to the modified surface and an in situ ELD is executed inside the microchannel to prepare the metal patterns on the surface. Drawing is not to scale. b) Photograph and micrograph (inset) of the PDMS microchannel stamp. c) Photograph and d) micrographs (inset in (c)) of the as‐made Cu pattern on PET.

Importantly, not only Cu structures but also other metals such as (Ag and Ni) were successfully fabricated on the PET substrates with the present method. To evaluate the performance of the in situ polymer‐assisted ELD in a microchannel, we fabricated a segment of the metal patterns (length 9.55 mm and width 138 µm). The morphology of the as‐made metal‐PET patterns was first characterized by scanning electron microscopy (SEM). Indeed, closely packed metal particles were formed in the patterned area as shown in **Figure** **2**. Energy‐dispersive X‐ray (EDX) spectroscopy further verified the successful fabrication of Cu, Ag, and Ni on PET substrates (Figure S4, Supporting Information). **Table** **1** summarizes the properties of the as‐made metals on PET substrates. By atomic force microscopy (AFM), the thickness of the Cu, Ni, and Ag patterns was determined to be 110 ± 11, 123 ± 14, and 106 ± 6 nm, respectively (Figure S3, Supporting Information). The as‐made metals exhibited a conductivity of 3.0 × 10^7^, 5.2 × 10^7^, and 1.0 × 10^7^ S m^−1^, for Cu, Ag, and Ni, respectively. To the best of our knowledge, the conductivity of the as‐prepared metal structures is among the best results on flexible substrates by ELD.

**Figure 2:**
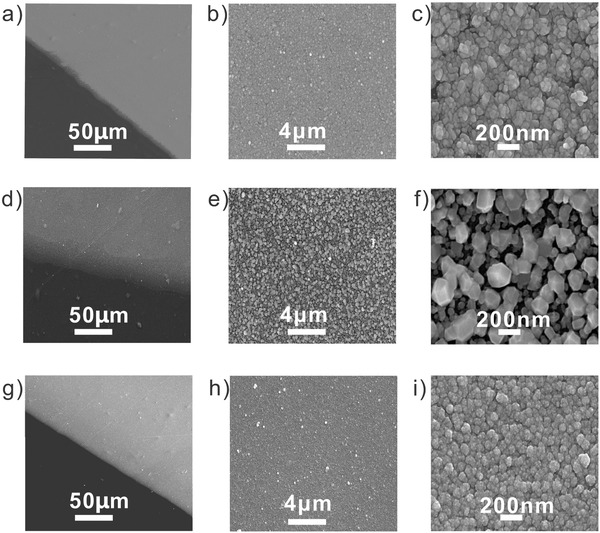
Field‐emission scanning electron microscopy(FE‐SEM) images of the as‐made metal structures patterned on PET substrates: a–c) Cu‐PET, d–f) Ag‐PET, and g–i) Ni‐PET.

**Table 1:** Summary of the properties of different metals on PET substrates

Metal‐PET	Thickness [nm]	Resistance [Ω]	Conductivity [S m^−1^]
Cu	110 ± 11	20 ± 3	3.0 × 10^7^
Ag	123 ± 14	11 ± 1	5.2 × 10^7^
Ni	106 ± 6	630 ± 90	1.0 × 10^7^

More importantly, the as‐made metal‐PET is highly flexible, while keeping a remarkable stability of the conductivity upon bending. In order to systematically evaluate the flexibility, we first investigated the resistance variation of various metal‐PET composites upon bending with decreasing radius of curvature (*r*) of the metal pattern (**Figure** **3**a). For the Cu‐PET sample, the normalized resistance (*R*/*R*_0_) remained stable (≈1.0) when *r* was larger than 0.75 mm. Here *R*_0_ depicts the resistance of unbent Cu interconnect. The *R*/*R*_0_ ratio slightly increased to 1.18 when *r* further decreased from 0.75 to 0.22 mm. For the Ag‐PET sample, the *R*/*R*_0_ remained unchanged for *r* larger than 5.99 mm. Gradually decreasing *r* from 3.11 to 0.22 mm results in an ≈2.7‐fold increase in normalized resistance. Similarly, for the Ni‐PET sample, the *R*/*R*_0_ ratio started to increase for radii smaller than 1.62 mm, until at a radius of 0.22 mm an ≈2.0‐fold normalized resistance was obtained. The increase of the resistance of metals at smaller bending curvatures could be attributed to crack formation in metal film, which are occurring during bending. These results are in good agreement with our previous findings.^29^

**Figure 3:**
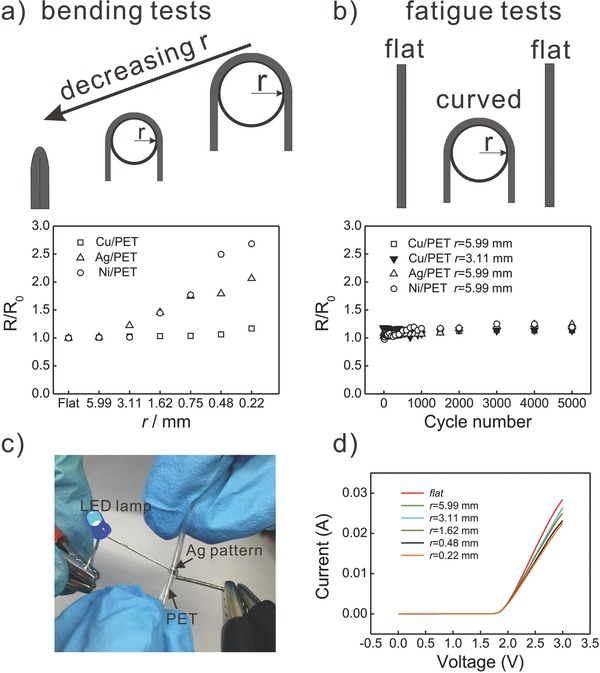
Electrical performance test of the as‐made metal‐PET. a) Normalized resistance of the Cu‐PET, Ag‐PET, and Ni‐PET at different bending radius. b) Fatigue tests of the resistance variations at different quantities of bending cycles. c) A bendable LED circuit, where an Ag‐PET pattern was used as an interconnect to light up an LED lamp. d) The *I–V* characteristics of the LED circuit (c) at different bending radii.

The as‐made metal interconnects also show excellent fatigue resistance at bending. The fatigue tests were carried out by bending each sample for 5000 cycles with a bending curvature of 3.11 or 5.99 mm, as shown in Figure 3b. We observed that the *R*/*R*_0_ remained stable (≈1.0) at the examined bending curvatures, indicating the mechanical durability of the as‐made metal‐patterned structures. As proof‐of‐concept for the application of the as‐made flexible metal interconnects, we demonstrated their application in flexible LED circuits to light up LED lamps at different bending curvatures (Figure 3c,d). The *I–V* characteristics of the circuits show some variation upon bending of the Ag‐PET interconnects, which are consistent with the previous bending tests.

In addition, the as‐made metal‐patterned structures show a remarkable durability for long‐term applications. **Figure** **4** shows the variation of the *R*/*R*_0_ at different storage time. For the Cu‐PET sample, the *R*/*R*_0_ gradually increased from 1.0 to 1.5‐fold as the storage time increased to 30 d, owing to the gradually oxidation of the coppers. For the Ag‐PET sample, the *R*/*R*_0_ increased to ≈1.5‐fold during the first 15 d and then remained unchanged for the next two weeks. For the Ni‐PET sample, the *R*/*R*_0_ was virtually constant during the experiments (30 d). Therefore, the as‐made metal‐PET interconnects are capable for long‐term applications.

**Figure 4:**
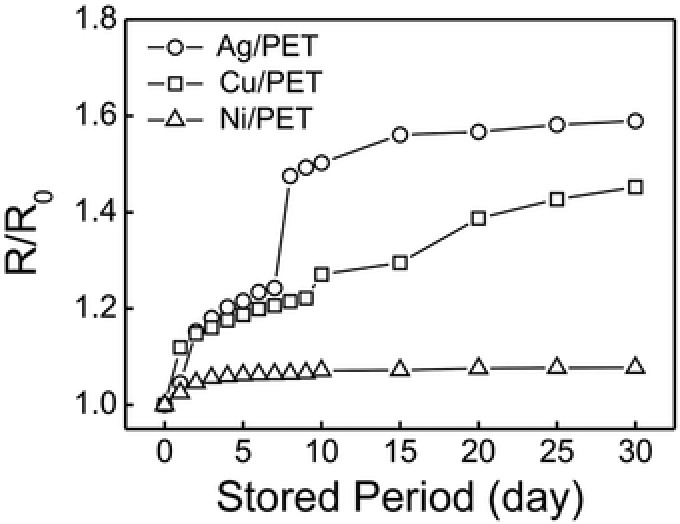
Dependence of the normalized resistance on the storage time of the as‐made metal‐PET interconnects.

In conclusion, we have developed a microfluidic approach for patterning of metals (e.g., silver, nickel, and copper) on chemically modified PET thin film surfaces by in situ polymer‐assisted ELD. With this method, flexible Cu‐PET, Ni‐PET, and Ag‐PET were successfully fabricated with extreme high electrical conductivity that is among the best of the metals prepared by ELD. This method has several remarkable advantages. First, a miniscule amount of catalyst solution or even recycled solution is required to conduct the loading of catalyst via the ion exchange by using the microfluidic approach. Second, the loading of metal plating solution as well as their plating process is similar to the catalyst loading executed in the microchannel. Therefore, both reagent consumption (expensive catalyst and metal solution) and reaction time are significantly decreased. Third, owing to the nature of microfluidics, structures of high‐quality metals with electrical conductivity comparable to the bulk metals were obtained in a much faster and lower cost pathway. However, the polymer‐assisted ELD process based on a microchannel approach has some limitation in fabricating arbitrary patterned structures of metals. Therefore, we envisage to resolve this issue applying some fluid pen‐based patterning techniques.^36^ Despite the current proof‐of‐concept demonstration via application of as‐made metals as the flexible interconnects for lighting up LED lamps, we envision that our low‐cost and versatile method is a good alternative to the previous surface patterning technique, concerning the fabrication of metal structures for flexible electronics.

## Experimental Section

*Materials*: PDMS prepolymer and curing agent (Sylgard 184) were purchased from Dow Corning. PET substrates (50 µm thick) are commercial products. 2‐methoxyethanol, ammonium tetrachloropalladate (II) ((NH_4_)_2_PdCl_4_), VTMS, METAC (80 wt% aqueous solution), KPS, and all other chemicals were purchased from Sigma‐Aldrich.

*Fabrication of the Stamp of the PDMS Microchannel*: The PDMS stamps were fabricated following the well‐established soft lithography process. Briefly, a thin layer of patterned photoresist on an Si wafer by photolithography was used as the master for the PDMS stamps. PDMS stamps were prepared using the Sylgard‐184 kit. The curing agent and the prepolymer were mixed in a 1:10 mass ratio and degassed before use. The mixture was then poured onto the master and cured at 65 °C for 2 h. Finally, the PDMS stamp was peeled off from the master, cleaned with ethanol, and dried with N_2_ flow.

*Fabrication of the PMETAC‐Modified PET Substrate*: PET substrates were cleaned with ethanol and then immersed in a 1 m NaOH solution for 40 min to clean the greasy on the surface. Afterward, PET substrates were then cleaned with DI water and dried by N_2_ . PET substrates were treated with air plasma for 5 min to generate hydroxyl groups on the surface. Subsequently, PET substrates were then immersed in a 2% (v/v) VTMS ethanol solution (pH was adjusted to 4) for 15 min to allow the reaction of silane with hydroxyl groups on the surface. The VTMS‐modified PET substrates were then immersed in a 20% (v/v) METAC aqueous solution for 60 min at 80 °C to carry out in situ free‐radical copolymerization on the substrate surface using KPS as an initiator. As a result, PMETAC‐modified PET substrates were obtained.

*Fabrication of the Metal‐PET Patterns*: A patch of PDMS microchannel was first reversibly attached to the as‐made PMETAC‐PET substrate. Then, a 4 × 10^−3^ m (NH_4_)_2_PdCl_4_ aqueous solution was infused into the microchannel for 3 min at a flow rate of 0.1 mL min^−1^ via a peristaltic pump. During pumping the [PdCl_4_]^2−^ catalytic ions were immobilized onto the quaternary ammonium groups of PMETAC polymer chains through ion exchange. Afterward, DI water was infused into the microchannel to rinse the physically adsorbed catalyst. Cu‐PET was fabricated by applying a typical Cu electroless plating solution in the microchannel for 2 min at a flow rate of 0.1 mL min^−1^ via a peristaltic pump.The Cu plating bath consisted of a 1:1 mixture of freshly prepared solutions A and B. Solution A contained NaOH (18.0 g L^−1^), CuSO_4_·5H_2_O (19.5 g L^−1^), and potassium sodium tartrate (43.5 g L^−1^) in DI water. Solution B was a formaldehyde (14.5 mL L^−1^) aqueous solution. The ELD of silver was conducted by first activating the PdCl_4_^2−^‐loaded substrate by applying the Cu plating solution through the microchannel for several seconds, followed by introducing Ag plating solution (Ag(NH_3_)_2_]NO_3_(1 g L^−1^) and potassium sodium tartrate (5 g L^−1^) aqueous solution) to the microchannel for 8 min at a flow rate of 0.1 mL min^−1^. The ELD of Ni was performed by introducing a plating solution consisting of Ni_2_SO_4_·5H_2_O (80 g L^−1^), sodium citrate (40 g L^−1^), lacticacid (20 g L^−1^), and dimethylamine borane (DMAB) (2 g L^−1^) in DI water for 3 min at a flow rate of 0.1 mL min^−1^. A Ni‐plating stock solution of all components except for DMAB reductant was prepared in advance. The ELD plating solution of Ni was prepared by mixing the Ni and reductant stock solution with a ratio of 4:1 v/v and adjusting the pH ≈ 8 with ammonia.

*Characterization*: The morphology and element analysis of metal‐PET patterns were investigated by SEM (JSM‐7800F&TEAM Octane Plus) coupled with EDX spectroscope.

AFM (multimode‐8, Bruker) was performed in noncontact mode to characterize the surface morphology and thickness of metal‐PET patterns. The resistance of metal‐PET patterns was measured using a four‐point probe method with a Keithley 2400 source meter. *I–V* characterization was measured using a Keithley 2400 Multimeter GPIB remote control.

## Supporting information

As a service to our authors and readers, this journal provides supporting information supplied by the authors. Such materials are peer reviewed and may be re‐organized for online delivery, but are not copy‐edited or typeset. Technical support issues arising from supporting information (other than missing files) should be addressed to the authors.

Supplementary
