# Supplementary material for: Microfluidic Patterning of Metal Structures for Flexible Conductors by In Situ Polymer‐Assisted Electroless Deposition
Source: Adv Sci (Weinh). 2016 Nov 1;4(2):1600313. doi: 10.1002/advs.201600313 (PMC5323856; doi:10.1002/advs.201600313)
Supplement: Supplementary file 1 — Supplementary [file ADVS-4-na-s001.pdf]

Copyright WILEY-VCH Verlag GmbH & Co. KGaA, 69469 Weinheim, Germany, 2016.

ADVANCED SCIENCE
Open Access

Supporting Information

for *Adv. Sci.,* DOI: 10.1002/advs.201600313

Microfluidic Patterning of Metal Structures for Flexible Conductors by In Situ Polymer-Assisted Electroless Deposition

*Suqing Liang, Yaoyao Li, Tingjiao Zhou, Jinbin Yang, Xiaohu Zhou, Taipeng Zhu, Junqiao Huang, Julie Zhu, Deyong Zhu, Yizhen Liu, Chuanxin He, Junmin Zhang, and Xuechang Zhou**

WILEY-VCH

Copyright WILEY-VCH Verlag GmbH & Co. KGaA, 69469 Weinheim, Germany, 2013.

Supporting Information

Microfluidic Patterning of Metal Structures for Flexible Conductors by in situ Polymer-Assisted Electroless Deposition

Suqing Liang,[1] Yaoyao Li,[1] Tingjiao Zhou,[1] Jinbin Yang,[1] Xiaohu Zhou,[2] Taipeng Zhu,[1] Junqiao Huang,[1] Julie Zhu,[1] Deyong Zhu,[1] Yizhen Liu,[1] Chuanxin He,[1] Junmin Zhang,[1] and Xuechang Zhou [1,]*

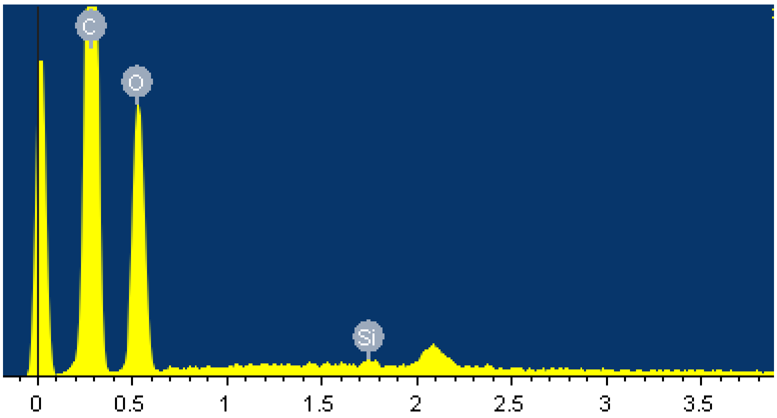

Elements	Weight%	Atom %
C K	58.37	65.17
O K	41.47	34.76
Si K	0.16	0.08

Figure S1. The EDX result of the as-made VTMS-PET. The result indicates the successful immobilization of VTMS on PET substrate by silanization process.

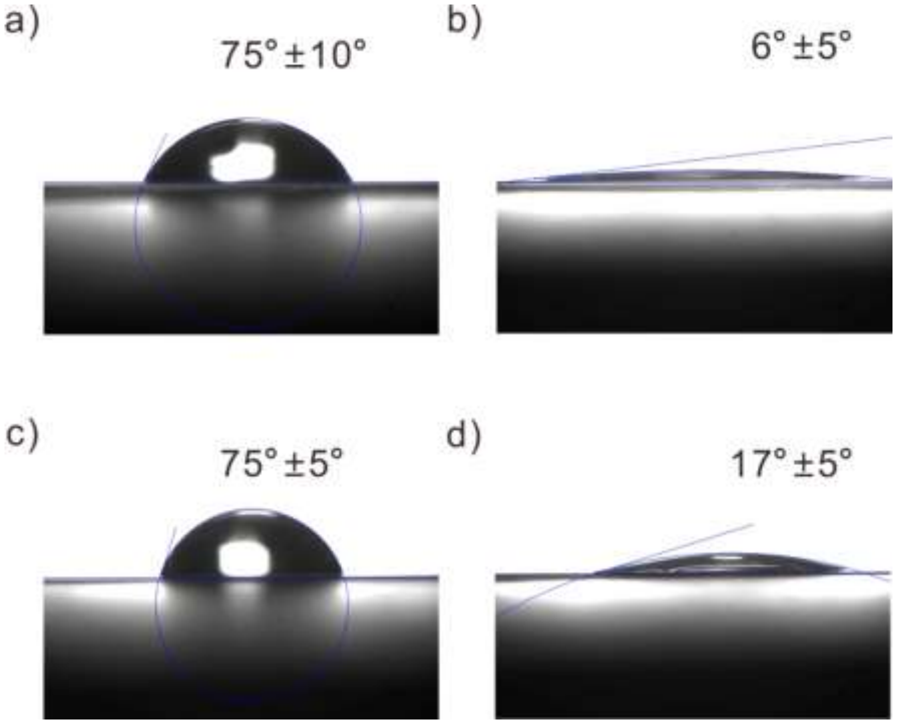

Figure S2. The water contact angle of the original PET (a), air plasma-treated PET (b), VTMS-PET (c), and PMETAC-PET (d) substrate.

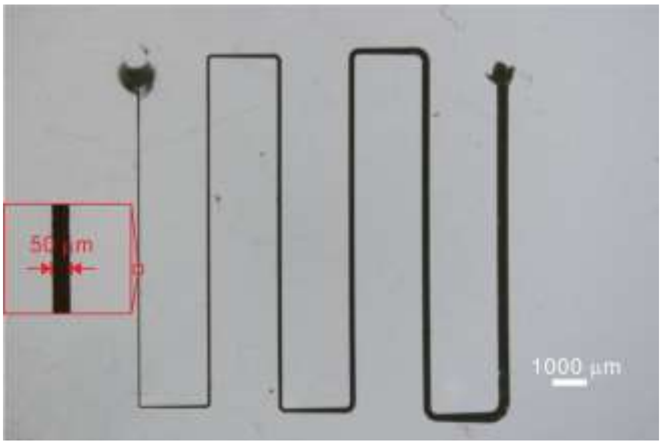

Figure S3. Optical micrograph of the as-made Cu-PET with width increasing from 50 to 250 μm. In the inset the thinnest of Cu-feature (50 μm) is shown.

a)

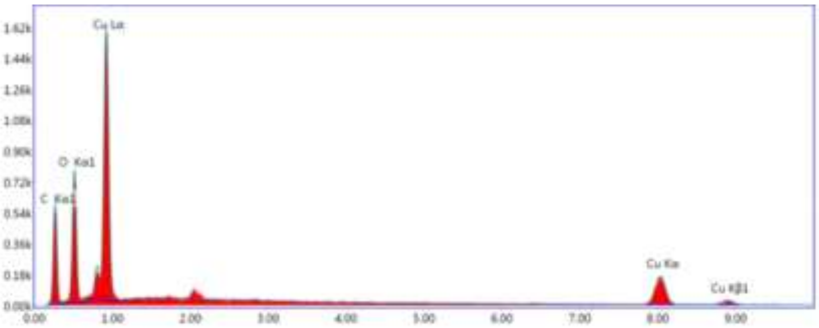

Intelligent quantitative results									
Element	Weight %	Atom %	Intensities	Error %	Kratio	Z	R	A	F
C K	30.47	54.50	222.34	9.51	0.11	1.17	0.91	0.31	1
O K	21.88	29.38	312.61	9.24	0.08	1.11	0.894	0.33	1
CuK	47.65	16.11	173.89	5.44	0.38	0.8	1.04	1.01	1

b)

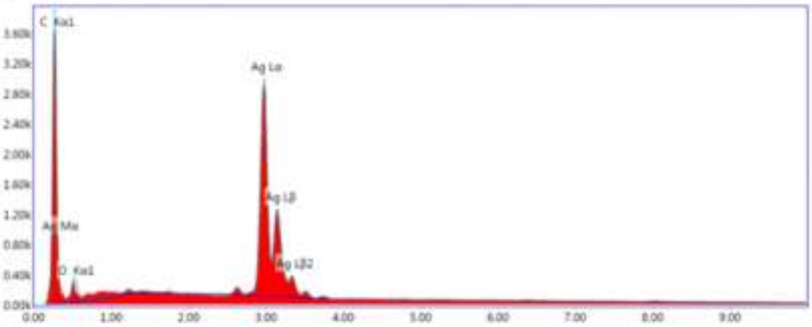

Intelligent quantitative results									
Element	Weight %	Atom %	Intensities	Error %	Kratio	Z	R	A	F
C K	33.72	72.49	1371.59	3.95	0.33	1.26	0.85	0.77	1
O K	8.48	13.68	115.46	12.67	0.01	1.2	0.88	0.14	1
AgK	57.80	13.84	1934.44	3.11	0.49	0.8	1.03	1.06	1

c)

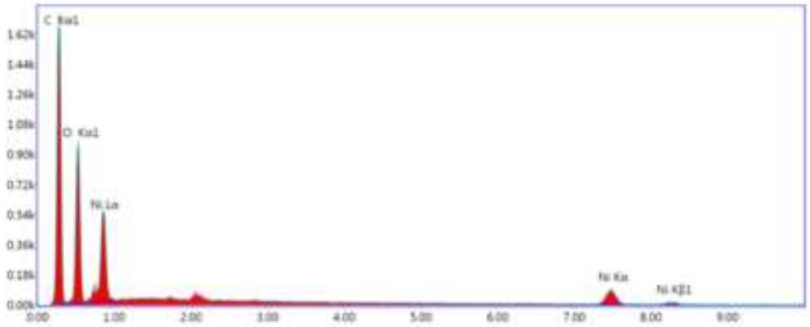

Intelligent quantitative results									
Element	Weight %	Atom %	Intensities	Error %	Kratio	Z	R	A	F
C K	51.51	65.30	637.52	6.98	0.27	1.06	0.97	0.5	1
O K	31.96	30.42	386.25	9.66	0.08	1.01	0.99	0.26	1
NiK	16.52	4.29	86.68	7.32	0.13	0.75	1.06	1.01	1

Figure S4. EDX results of the as-made metal-PET composite: a) Cu-PET, b) Ag-PET, and c) Ni-PET.

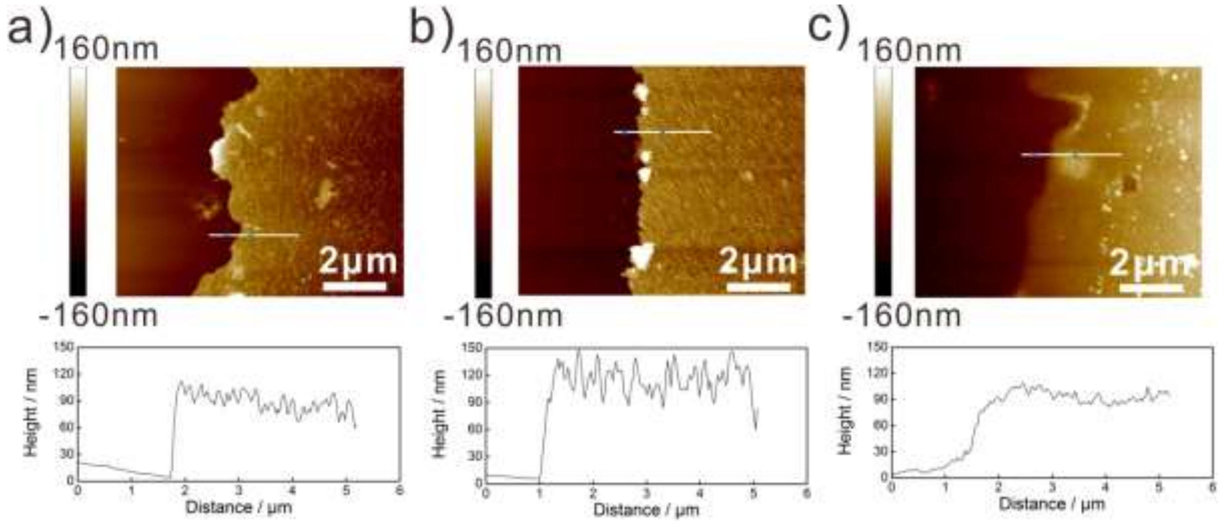

Figure S5. AFM topographic images of the as-made metal-PET and cross-sectional profiles: a) Cu-PET, b) Ag-PET, and c) Ni-PET.

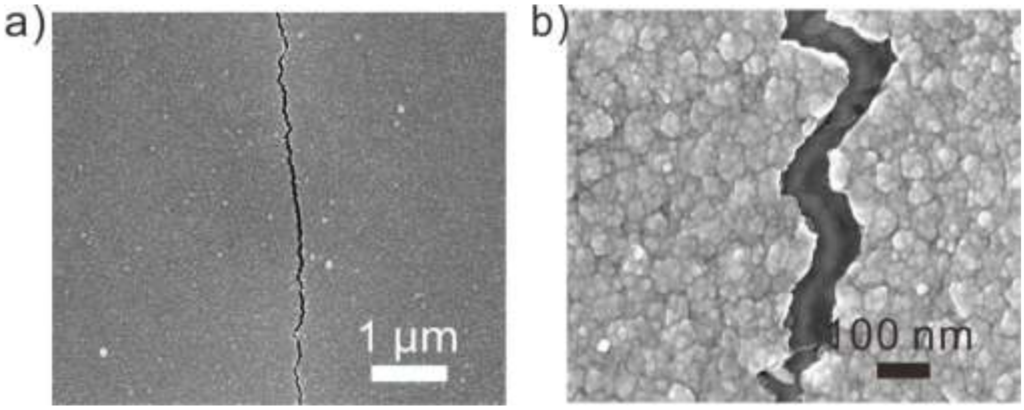

Figure S6. SEM images of the Cu-PET after 5000 times of bending cycles with a bending curvature of 3.11 mm. Here the dark areas depict cracks of the metal film.
